# Chemokines and Chemokine Receptors: Accomplices for Human Immunodeficiency Virus Infection and Latency

**DOI:** 10.3389/fimmu.2017.01274

**Published:** 2017-10-16

**Authors:** Zhuo Wang, Hong Shang, Yongjun Jiang

**Affiliations:** ^1^Key Laboratory of AIDS Immunology of National Health and Family Planning Commission, Department of Laboratory Medicine, The First Affiliated Hospital, China Medical University, Shenyang, China

**Keywords:** reservoir, inflammation, immune activation, resting, CD4, T cell, disease progression, cure

## Abstract

Chemokines are small chemotactic cytokines that are involved in the regulation of immune cell migration. Multiple functional properties of chemokines, such as pro-inflammation, immune regulation, and promotion of cell growth, angiogenesis, and apoptosis, have been identified in many pathological and physiological contexts. Human immunodeficiency virus (HIV) infection is characterized by persistent inflammation and immune activation during both acute and chronic phases, and the “cytokine storm” is one of the hallmarks of HIV infection. Along with immune activation after HIV infection, an extensive range of chemokines and other cytokines are elevated, thereby generating the so-called “cytokine storm.” In this review, the effects of the upregulated chemokines and chemokine receptors on the processes of HIV infection are discussed. The objective of this review was to focus on the main chemokines and chemokine receptors that have been found to be associated with HIV infection and latency. Elevated chemokines and chemokine receptors have been shown to play important roles in the HIV life cycle, disease progression, and HIV reservoir establishment. Thus, targeting these chemokines and receptors and the other proteins of related signaling pathways might provide novel therapeutic strategies, and the evidence indicates a promising future regarding the development of a functional cure for HIV.

## Introduction

Chemokines are low-molecular-weight proteins that belong to the cytokine superfamily and induce immune cell trafficking by binding to their corresponding receptors (1). Currently, chemokines are classified into four major subfamilies: CXC, CC, XC, and CX_3_C, and 17 CXC chemokines, 28 CC chemokines, 2 XC chemokines, 1 CX_3_C chemokine, and approximately 20 chemokine receptors have been found (2–5). Most chemokines exert their biological functions by binding to chemokine receptors, which are G protein-coupled receptors (GPCRs) with seven transmembrane domains, to promote cell survival and proliferation and act as guides for cell homing and migration (6). In addition to their most highly recognized roles in cell migration, these small chemoattractant molecules have multiple other functional properties (7). Chemokine expression increases when there is tissue damage, and most chemokines are recognized as pro-inflammatory factors; they have been shown to exert regulatory functions in a wide range of pathological and physiological contexts, such as hypersensitivity reactions, infection, angiogenesis, inflammation, tumor growth, and hematopoietic development (3, 8, 9). Given their critical roles in inflammation, many chemokines and chemokine receptors have been identified as potential therapeutic targets in a wide range of inflammatory diseases (10).

Human immunodeficiency virus (HIV) infection severely impacts the host immune system in many ways, such as causing the specific loss of CD4^+^ T cells, elevated immune activation and inflammation, and dysfunction of multiple immunocytes (11). During chronic HIV infection, impairment of the integrity of the gastrointestinal mucosa results in the microbial translocation, which is a possible cause of chronic systemic immune activation (12, 13). Accompanied by aberrant activation of the immune system, pro-inflammatory cytokines (including chemokines) are upregulated and are associated with HIV disease progression and mortality (14, 15).

What is more, during the early phase of infection, HIV may induce increased cytokine secretion (including chemokine secretion) *via* the innate immune response, promote immune activation and lead to a “cytokine storm” (16, 17). Ndhlovu et al. showed that immune activation occurs within 1–3 days of hyperacute HIV infection, and the “cytokine storm” can be observed before the peak viremia (16, 18). Multiple kinds of cytokines (including chemokines) have been shown to be elevated in the “cytokine storm,” such as interleukin (IL)-15, interferon (INF)-α, CXCL10 (known as INF γ-induced protein 10, IP-10), IL-8, and fractalkine (16, 19, 20). For instance, the chemokine CXCL10 is significantly elevated in 100% of HIV-infected individuals during early HIV infection and impacts on the subsequent disease progression (16, 21–23). Also, IL-8 (CXCL8) is elevated in acute HIV infection, but more slowly than CXCL10 (16), and it has been reported that high IL-8 concentrations in the genital tract are correlated with a low CD4^+^ T cell count during acute HIV infection (24). Irrespective of whether the infection is in the acute or chronic phase, the levels of many chemokines are upregulated, and the expression of chemokine receptors is altered. What is the effect of these changes on viral replication, CD4^+^ T cells depletion, immune function, disease progression, and HIV reservoir establishment? All these issues need to be reviewed.

The goal of this review was to summarize current knowledge from recent studies that have identified novel roles of chemokines during HIV infection and latency and provide an insight into the signaling mechanisms of chemokines and their receptors, highlighting potential therapeutic targets, and helping to frame the current and future immune therapy approaches.

## Chemokines and Chemokine Receptors Related to HIV Replication and Disease Progression

Recently, researchers have reported that chemokines and chemokine receptors play critical roles in viral infection. Alterations of chemokine concentrations and chemokine receptor expression contribute to persistent immune activation, which further impacts on the life cycle of HIV and subsequent disease progression. Here, we summarize the chemokines and chemokine receptors associated with HIV replication and disease progression.

### CXCR4 and CCR5

Both CXCR4 and CCR5 are GPCRs. CXCR4 is specifically activated by chemokine CXCL12 (stromal cell-derived factor 1) and participates in physiological activities such as chemotaxis, cell proliferation and survival, and intracellular calcium flux (25, 26). Natural ligands for CCR5 include CCL3 (MIP-1α), CCL4 (MIP-1β), CCL5 (RANTES), CCL8 (MCP-2), CCL11 (eotaxin), CCL14 (HCC1), and CCL16 (HCC4) (27, 28). CCR5 interacts with its ligands to regulate chemotaxis and cell activation (27). The HIV envelope glycoprotein (gp120) binds to the target cell by interacting with CD4 molecules with high affinity, but it is not sufficient for HIV entry. In the post-binding stage, CXCR4 or CCR5, acting as a co-receptor with CD4, is necessary for the fusion of the viral envelope with the cell membrane (29, 30). CXCL12 and CCL5, which are ligands for CXCR4 and CCR5, respectively, can competitively inhibit HIV infection (31, 32). CXCR4 was the first reported HIV co-receptor; it was identified in 1996, the same year that CCR5 was identified as another co-receptor for HIV entry. The identification of the two co-receptors dramatically accelerated the exploration of HIV physiology and pathogenesis and laid the foundations for new therapeutic and preventive strategies (33).

CCR5 is the predominant receptor for the entry of CCR5-tropic viruses into cells, and lack of the CCR5 receptor on the cell surface has been reported to provide natural resistance against HIV transmission, which led to the functional cure of the “Berlin patient” (34–36). The “Berlin patient” went into remission, with no detectable viral load, due to the transplantation of bone marrow from a CCR5 delta32 (Δ32) homozygous donor whose CCR5 gene had a 32-bp deletion. This led to the production of a non-functional gene product, so CCR5 receptors could not be expressed on the cell surface (36). The case of the “Berlin patient” provides evidence that targeting the co-receptor CCR5 to eliminate HIV is possible (37), and so this approach is being recognized as a new treatment strategy. Accordingly, the CCR5 receptor antagonists such as maraviroc and cenicriviroc have emerged as new entry inhibitors (38, 39), and CCR5-targeting drugs have exhibited excellent potency and low toxicity in clinical trials (40). In spite of the fact that strictly CCR5-tropic viruses are present among nearly all founder and transmitter virus populations, the viruses in approximately 50% of HIV-1 subtype B patients will spontaneously develop into CXCR4-tropic viruses as the disease progresses, and the presence of CXCR4-tropic viruses is associated with worse clinical prognosis (41).

Accordingly, as an HIV infection progresses, the viral tropism usually changes to include more CXCR4 tropism, so the CXCR4 receptor gradually plays increasingly vital roles, especially in the entry process. As a consequence, CXCR4 provides an alternative approach for combating HIV (38). At present, several kinds of CXCR4 antagonists have been reported. One type is non-peptide small-molecule antagonists such as AMD3100, AMD3465, and AMD070. AMD070 has been assessed in a phase II trial due to its better oral bioavailability, safety, and the fact that it is well tolerated. Another kind of CXCR4 antagonist is peptide analogs, such as isothioureas, KRH, indole, piperidine, and purine CXCR4 antagonists (42–46). However, there is still a long way to go before CXCR4 antagonists are used in clinical treatment.

### CXCL10 and CXCR3

C–X–C motif chemokine 10 (CXCL10) is also known as IP-10 (47, 48). The biological function of CXCL10 is to induce chemotaxis, cell growth, angiogenesis, and apoptosis by binding to its surface chemokine receptor CXCR3, and CXCL10 is recognized as an inflammatory chemokine (49). Moreover, CXCL10 plays an important role in various pathological states. CXCL10 had been reported to act as a marker for the diagnosis of tuberculosis (50, 51) and hepatitis B (52, 53) and to promote tumor progression, such as in pancreatic cancer progression (54). Recently, studies have shown that CXCL10 also plays a role in HIV infection. Jiao et al. revealed that CXCL10 was the only cytokine, among 26 cytokines, that was significantly elevated during the early stages of HIV infection, and it was positively associated with disease progression (21). It has been demonstrated that CXCL10 levels were significantly increased in untreated HIV-infected patients and could not be reduced to normal levels by antiretroviral therapy (ART); moreover, persistently high levels of CXCL10 are associated with immunological treatment failure following ART in HIV-infected patients (55, 56). In HIV-exposed seronegative sex workers, significantly lower expression of CXCL10 has been associated with strong protection of the mucosal immune system against HIV infection (57). Also, high systemic CXCL10 levels before infection were revealed to be associated with rapid HIV progression, and the level of systemic CXCL10 during primary HIV infection is positively associated with HIV DNA levels and viral load and negatively associated with CD4^+^ T cell count (22, 56).

CXCR3 is a GPCR with seven transmembrane-spanning domains, the ligands of which include CXLCL9 (MIG), CXCL10 (IP-10), and CXCL11 (I-TAC). Foley et al. demonstrated that CCR5^+^CD4^+^ T cells express CXCR3 receptors, which could be recruited by CXCL10 and CXCL11 to local HIV-infected lymph nodes (23). This recruitment may enhance the retention of T cells in HIV-infected lymphoid organs, which leads to sustained disruption of the peripheral T cell response and elevated T cell susceptibility to HIV due to prolonged exposure to high viral concentrations, contributing to the immunopathology of acquired immunodeficiency syndrome (23).

### CCL19/CCL21 and CCR7

The chemokines CCL19 and CCL21 are abundant in lymphoid organs, and they regulate the homing of naïve and central memory T cells to lymph nodes by binding to the receptor CCR7 (58, 59). However, they activate different signal transduction pathways. CCL21 mediates the majority of migratory events (through CCR7), whereas CCL19 seems to play a supplementary role in migration and provides additional signals such as promoting cell survival (60). Despite the fact that CCL19 and CCL21 are mainly produced in secondary lymphoid tissue, CCL19 and CCL21 production in non-lymphoid organs has been observed during inflammatory and infectious diseases (61, 62). In HIV-infected individuals, elevation of plasma CCL19 has been demonstrated during acute HIV infection (0–3 months), chronic HIV infection (24 months), and after ART use for 9–12 months (63). Damås et al. reported that serum levels of CCL19 and CCL21 positively correlate with plasma HIV RNA levels and negatively correlate with CD4 cell count (59). They also observed that CCL19 and CCL21 were sustained at high levels among patients who were virologic non-responders to ART. Moreover, the levels of CCL19 and CCL21 were lower in patients with no HIV disease progression compared with those with disease progression (59). Therefore, dysregulation of CCL19 and CCL21 levels during HIV infection might profoundly influence HIV disease progression and treatment.

CCR7, the receptor for CCL19 and CCL21, is expressed at high levels on central memory and naïve T cells, and it plays a vital role in the homing of T cells to the peripheral lymphoid organs. CCR7 is one of the most prominent chemokine receptors in the adaptive immune system (58). Hayasaka et al. demonstrated that gp120-induced CXCR4 signaling can upregulate CCR7 function to promote CCR7-dependent CD4^+^ T cell migration, possibly by promoting CCR7 homo- and CXCR4/CCR7 hetero-oligomers formation on the surface of CD4^+^ T cells (64).

However, most other researchers hold that CCR7 is downregulated in HIV infection. Ramirez et al. reported that HIV viral protein U can downregulate CCR7 on CD4^+^ T cells, which impairs their migration toward CCL19 (65). Similarly, Perez-Patrigeon et al. demonstrated that, in HIV-infected subjects, the proportion of CCR7^hi^ T cell subsets is decreased, and, in viremic patients, CCR7-dependent chemotactic responses are significantly decreased (66). Impairment of CCR7-induced migration might indirectly promote HIV infection by prolonging the duration of the CD4^+^ T cell presence in peripheral tissue, such as mucosal tissue, which may have a high virus titer and productively infected cells (66). On the other hand, CCR7 downregulation also impairs cell migration to the peripheral blood, thereby disrupting peripheral immune function.

### C–C Motif Chemokine Ligand 20 (CCL20) and CCR6

C–C motif chemokine ligand 20, which is also known as liver and activation-regulated chemokine and macrophage inflammatory protein-3α, has a strong chemotactic effect on immature dendritic cells (DCs) and lymphocytes, and weakly attracts neutrophils (67, 68). Lee et al. and Baba et al. suggested that CCL20 was upregulated during inflammation and elicited its effects on its target cells by binding to and activating the chemokine receptor CCR6 (69, 70). During HIV infection, CCL20 has been shown to remain elevated throughout the course of the disease; the median CCL20 serum level was approximately 3.3-fold higher in HIV-infected individuals than in uninfected individuals and was negatively correlated with CD4^+^ T cell count (63, 71).

Further study showed that CCL20 is constitutively produced by the primary epithelial cells of female genital organs, and it is upregulated when female genital organs are exposed to healthy seminal plasma, which acts as an inflammatory factor (72). Elevated CCL20 further enhances the recruitment of Langerhans cell precursors, which are permissive to HIV infection (73). In addition, saliva is a complex cocktail that is composed of multiple physiologic molecules, including chemokines (74, 75), and CCL20 in saliva might also contribute to sexual transmission of HIV during oral sex (76). These findings indicate that CCL20 might accelerate heterosexual transmission of HIV.

During the acute and posttreatment phases of HIV infection, the significantly increased plasma CCL20 may be a reason for the low DC level in circulating blood due to recruitment of myeloid dendritic cells to peripheral sites to fight the virus (63). In a simian immunodeficiency virus (SIV)-macaque model, CCL20 was found to be released from epithelial cells after vaginal exposure to SIV. CCL20 might recruit CCR6^+^ plasmocytoid dendritic cells (pDCs) to the region just beneath the cervical epithelium and then produce cytokines, such as IFN-α, CCL3, and CCL4. These molecules are capable of recruiting CCR5^+^CD4^+^ T cells, which are highly susceptible to SIV infection, to the local area (77, 78). These data suggest that CCL20 can promote SIV infection indirectly. However, other researchers hold opposite opinions. Ghosh et al. reported that when primary uterine and fallopian tube tissues were treated with CCL20 and HIV at the same time, HIV infection was inhibited, but these results were not observed when CCL20 was added before or after infection (79). Their data indicate that CCL20 has an endogenous anti-HIV effect on the female reproductive tract. However, they did not reveal the exact mechanism of this phenomenon. Similarly, Shang et al. demonstrated that in the genital tract epithelia of non-human primates, the pDCs recruited by CCL20 can release IFN-α and CCL4, which might inhibit viral entry but fail to control the infection (77). Although the effect of CCL20 on HIV infection is still controversial, most studies support the theory that CCL20 promotes HIV infection and disease progression.

CCR6 (CD196), a receptor for CCL20, is mainly expressed on memory T cells, DCs, some B cell subsets, natural killer (NK) cells, and γδ T cells (80–82). In addition to mediating chemotaxis involving CCR6-expressing cells, it may act as an important target of HIV. CCR6^+^ T cells, including memory, TH_17_ and α4β7^+^ T cells, have been shown to be highly susceptible to HIV infection (83). Recently, CCR6 has been identified as a weaker independent co-receptor for the HIV entry process, but this has only been confirmed in the HP-2 cell line in an *in vitro* study (84), and no evidence of CCR6 acting as an HIV co-receptor has been demonstrated *in vivo* yet. Human β-defensin 2 (hBD2) is also a ligand of CCR6, and hBD2 binding to CCR6 has been shown to confer direct anti-HIV activity (85). Moreover, hBD2-induced CCR6 activation has also been found to directly inhibit HIV infection during the postentry phase, through apolipoprotein B mRNA-editing enzyme-catalytic polypeptide-like 3G, which causes interference with HIV reverse transcription (83). These observations indicate that selectively targeting CCR6^+^ cells may serve as a novel prevention and treatment strategy in the future.

### C–C Motif Chemokine Ligand 2 (CCL2) and CCR2

C–C motif chemokine ligand 2, which is also referred to as monocyte chemoattractant protein 1, binds to its receptor, CCR2, and plays multiple physiological roles. CCL2 is produced by several types of cells, and monocytes/macrophages are the major source among leukocytes (86, 87). CCL2 is a chemoattractant for CD4^+^ T cells, monocytes/macrophages, and NK cells, recruiting them to the sites of infection and inflammation. At the same time, CCL2 is an important factor during monocyte differentiation to macrophages (88). Macrophages play an important role in the pathogenesis of HIV-1 infection (89), as indicated by the fact that HIV has been shown to be able to replicate in human macrophages *in vitro* (90, 91).

As CCL2 is an inflammatory chemokine, the CCL2/CCR2 axis has been suggested to be involved in HIV-associated neurologic disorders (92, 93). Several researchers have reported an upregulation of plasma CCL2 and its transcript levels in HIV infection (94–96). In addition, it has been observed that CCL2 is elevated during HIV infection, and it promotes viral replication in infected macrophages (97, 98). Moreover, neutralizing CCL2 using antibodies restricts HIV replication in macrophages, suggesting that CCL2 is involved in a postentry process of the viral life cycle, rather than an entry process. Interestingly, researchers have further illuminated that CCL2 is negatively associated with apolipoprotein B mRNA-editing enzyme-catalytic polypeptide-like 3A expression, which helps to unravel the possible mechanism of CCL2’s effect on HIV infection (98). In the context of HIV infection, in addition to its role in cell migration and inflammation, CCL2 directly promotes viral replication in T lymphocytes, peripheral blood mononuclear cells, and macrophages (98–100). Campbell and Spector identified that when resting CD4^+^ T cells were exposed to CCL2, upregulation of CXCR4 expression occurred, and the elevated CXCR4 expression increased infection by CXCR4-tropic HIV (100). These results suggest that HIV infection upregulates CCL2 gene expression and secretion, and CCL2 may in turn represent an important factor that enhances HIV spread and infection.

## Chemokines and Chemokine Receptors Play Critical Roles in HIV Latency

Although the control of HIV replication by ART allows the immune system to be partially restored and delays disease progression, curing HIV infection still remains unachievable with the currently available ART drugs. HIV latency is the major obstacle to achieving a “sterilizing” cure. It is known that resting memory CD4^+^ T cells, monocytes, and some tissue cells can act as “shelter” for HIV. Among them, resting memory CD4^+^ T cells are the most important reservoir, because of their resting, homeostatic proliferation and long-lived characteristics. The infection level in resting memory CD4^+^ T cells, involving HIV entry and integration, might determine the size of the viral reservoir. Once an HIV reservoir has been established, it is difficult to eliminate using current treatment strategies. As mentioned earlier, chemokines and chemokine receptors play important roles in HIV replication and disease progression, and next we will discuss their effects on the HIV reservoir and new ways to clear the HIV reservoir.

### CCL19 and CCL21

CCL19 and CCL21 are constitutively expressed chemokines in lymphoid organs, and they have been demonstrated to be associated with HIV infection and disease progression (as mentioned earlier). As is well known, resting memory CD4^+^ T cells are the major constituent cells involved in HIV reservoirs, and they mainly reside in and travel between secondary lymphoid tissues, in which resting memory CD4^+^ T cells are more efficiently infected by HIV than those in the blood (101). Furthermore, CCR7-expressing resting CD4^+^ T cells are the main subset latently infected with HIV (102, 103).

Therefore, the levels of CCL19 or CCL21 (ligands for CCR7) in both peripheral and secondary lymphoid tissues might impact on the interaction between HIV and resting memory CD4^+^ T cells. Damås et al. found that HIV-infected patients with advanced immunodeficiency had higher serum CCL19 levels, and elevated CCL19 levels were associated with higher mortality (104). Moreover, Saleh et al. and Cameron et al. suggested that CCL19 and CCL21 contribute greatly to HIV latency in resting CD4^+^ T cells by promoting HIV entry and integration (101, 105). Similarly, Anderson et al. revealed that CCL19 enhanced both T- and M-tropic viral latency establishment in resting CD4^+^ T cells, and the M-tropic virus was more efficient (106). Regarding the related signaling mechanism, Saleh et al. suggested nuclear factor-κB signaling was involved in HIV reservoir establishment (107). These studies have provided novel insights into the effect of chemokines and receptors on HIV latency.

### CXCL12 and CXCR4

CXCL12 is the natural ligand of CXCR4, and CXCR4 is a co-receptor for T-tropic HIV infection. The physiological function of CXCL12 is to bind to CXCR4, driving progenitor cells to migrate to the bone marrow, and it can also cause cells to migrate to peripheral tissues during certain pathological conditions (108). To some degree, activation of the signaling pathway mediated by gp120/CXCR4 mimics the signal transduction mediated by CXCL12/CXCR4 (109). Both CXCL12 and gp120 can combine with CXCR4 to induce signaling in memory CD4^+^ T cells, and further promote the activation of cortical actin. As a result, there is a competition between HIV gp120 and CXCL12 for the CXCR4 receptor (32, 110). It has been proved that gp120 causes impairment of the T cell response to CXCL12-induced chemotaxis, whereas CXCL12 inhibits infection by CXCR4-tropic virus (32, 111). In this way, CXCL12 might inhibit CXCR4-tropic infection of memory CD4^+^ T cells and further inhibit the establishment of HIV latency.

According to these findings, CXCL12 and CXCR4 are regarded as targets for new methods of inhibiting CXCR4-tropic HIV infection of resting CD4^+^ T cells. Guo et al. found that a tyrosine kinase inhibitor, genistein, could efficiently inhibit HIV infection of resting CD4^+^ T cells, while being harmless to cells at experimental concentrations (110, 112). HIV infection of resting CD4^+^ T cells is also inhibited by sunitinib, another tyrosine kinase inhibitor, which is used as an antineoplastic drug in clinical settings (110). By inhibiting HIV infection of resting CD4^+^ T cells, these drugs may have the ability to reduce the HIV reservoir size at the beginning of the infection. These findings suggest that additional related methods could be developed to cure HIV.

### CXCR3

Given the high systematic CXCL10 levels during the early phase of HIV infection, and the fact that CXCR3 expression might indirectly promote HIV infection (as mentioned earlier), the relationship between CXCR3^+^ cells and the HIV reservoir needs further exploration. Khoury et al. demonstrated that the frequency of cells harboring integrated HIV DNA was positively associated with CXCR3^+^ expression on memory CD4^+^ T cells, irrespective of whether these cells were CCR6 positive or negative (113). Meanwhile, a subset of CD4^+^ T cells co-expressing CXCR3 and CCR6 become preferentially enriched for HIV DNA in HIV-infected ART-treated individuals (113). Similarly, Gosselin et al. identified CXCR3^+^CCR6^+^ T cells to be highly permissive to HIV replication (114). In conclusion, CXCR3 might act as a marker of the HIV reservoir and may be useful as a target for reservoir elimination.

## Possible Mechanisms of Chemokine-Related Actin Activation in the Promotion of HIV Infection and Establishment of Latency

**Figure 1:**
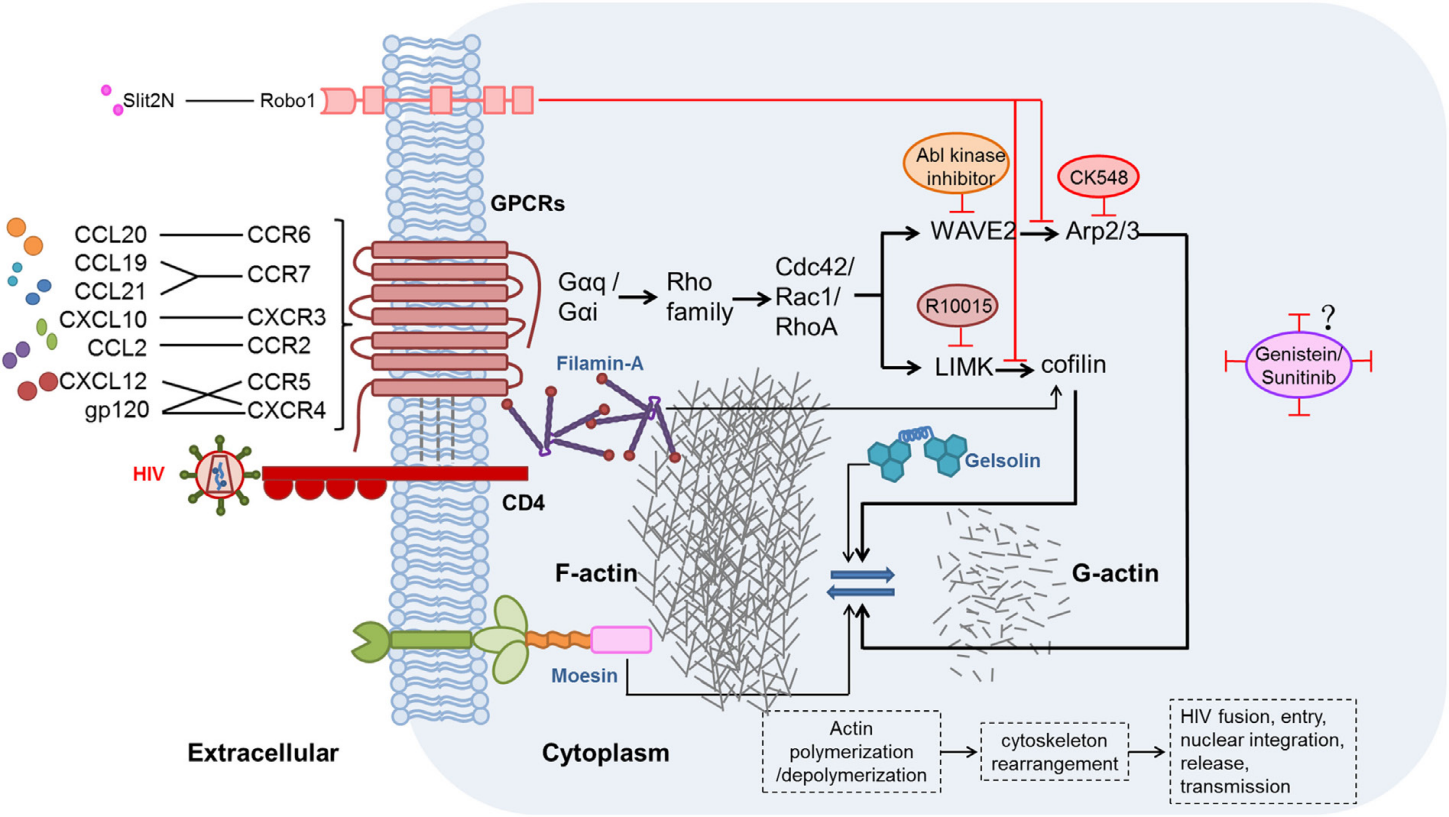
Possible mechanisms and signaling pathways of chemokines, chemokine receptors, and other proteins affecting the modulation of actin during human immunodeficiency virus (HIV) infection. HIV gp120 binding to the CXCR4 or CCR5 co-receptor, thereby mimicking the CXCL12/CXCR4 interaction, induces actin-related signaling. In addition, CXCL10, CCL19, CCL21, C–C motif chemokine ligand 20 (CCL20), and C–C motif chemokine ligand 2 (CCL2), which are elevated during HIV infection, bind to their receptors and thereby activate actin-related signaling (56, 69, 100, 105). Moesin (118, 119), filamin-A (120), and gelsolin (121) also promote actin-related signaling pathways. Two major actin-related signaling pathways, such as LIMK1–cofilin (122) and WAVE2–Arp2/3 (123), induce polymerization and depolymerization of actin, further leading to rearrangement of the cytoskeleton, and thus benefit HIV fusion, entry, nuclear integration, release, and, ultimately, transmission. By contrast, Slit2N binding to Robo1 (124) could inhibit the two major actin-related signaling pathways. R10015 (125), CK548 (126), Abl kinase inhibitor (127), genistein (110, 112), and sunitinib (110) can also inhibit actin-related signaling.

The effects of chemokines and their receptors on HIV replication and latency have been demonstrated, but studies of the related mechanisms are limited. During hyperacute HIV infection, pro-inflammatory cytokines (including chemokines) are upregulated before the peak viremia, and concurrently, a proviral reservoir is quickly established within days of the acute HIV infection (115, 116). Chemokines interacting with their related receptors induce actin activation, which can promote HIV entry and integration in resting CD4^+^ T cells (105, 117). The activation state of actin is regulated by many factors, including two signaling pathways discussed here. These pathways might be valuable targets for actin regulation and combating HIV infection. The possible mechanisms of chemokine-induced actin activation in the promotion of HIV infection and establishment of latency are illustrated in Figure 1.

### Actin Plays Crucial Roles in HIV Infection and Establishment of Latency

As a major intracellular component, actin has functions related to cell motility, cell signaling, and maintenance of cell junctions and cell shape. Moreover, the relatively static filamentous actin (F-actin) forms dense network structures, acting as a physiological barrier to pathogens (117). For this reason, it seems that it would not be easy for pathogens to transfer from the cell surface to the nucleus after infection. Likewise, cortical actin is the first barrier that HIV encounters during the entry and integration processes. The barrier in resting memory CD4^+^ T cells is much stronger than that in active cells, which have high levels of actin depolymerization (117).

To overcome this barrier, HIV gp120, imitating the chemokine CXCL12, binds to the co-receptor CXCR4 or CCR5 on resting memory CD4^+^ T cells, which leads to the activation of actin and allows the virus to break through the barrier, promoting viral entry, early DNA synthesis, and nuclear migration (122, 128). It has been found that signal transduction involving the chemokine co-receptor CXCR4, but not CD4, is required for HIV latent infection of resting T cells (117). Regarding CXCR4-tropic HIV, CXCR4 has been reported to be an absolute requirement for infection of resting CD4^+^ T cells purified from HIV-positive patients’ blood (117). Without the interaction of CXCR4 and gp120, actin could not be activated to enhance the HIV infection, and therefore, the activation of actin is crucial for the establishment of the HIV infection.

Although HIV gp120 could interact with its co-receptors (CXCR4 or CCR5) to depolymerize/polymerize actin, we should not ignore the fact that chemokines binding to their receptors also induce signaling that causes actin depolymerization/polymerization. Therefore, some chemokines might also contribute to the promotion of HIV infection, transmission, and reservoir establishment. As reported by Cameron et al., CCL19 and CCL21 modulate HIV entry and integration *via* their effect on cytoskeletal rearrangement and cortical actin activation; when actin depolymerization was inhibited, decreased HIV DNA integration in CCL19-treated resting CD4^+^ T cells was observed (105). Besides these two chemokines, it is speculated that other chemokines might also active actin after binding to their receptors and enhance the infection efficiently.

### Effect of Signaling Pathways on Actin Regulation

To date, two signaling pathways have been reported to be associated with actin activation: the LIMK1–cofilin pathway (122) and the WAVE2–Arp2/3 pathway (123). In the LIMK1–cofilin signaling pathway, cofilin and LIMK1 are the two major factors that regulate actin. Cofilin is an actin-binding protein; it is also known as the cortical actin-depolymerizing factor, as it depolymerizes actin filaments (129). In resting CD4^+^ T cells, the primary form of cofilin is phosphorylated cofilin, which is inactive. However, once HIV gp120 binds to a chemokine receptor on the resting CD4^+^ T cells, cofilin is dephosphorylated and becomes active within a few minutes, and it subsequently induces actin depolymerization (117, 130). The initiation of this signaling pathway ensures that HIV accomplishes its life cycle, including virus fusion, entry, reverse transcription, integration, and subsequent transcription. It has been demonstrated that when resting CD4^+^ T cells were treated with CCL19, cofilin phosphorylation and corresponding actin dynamics were observed within minutes, which further promoted HIV DNA integration (105). LIM domain kinase (LIMK) has been reported to be a kinase that phosphorylates and thereby inactivates cofilin (129). In recent years, LIMK was reported to be involved in the process of HIV infection, because cofilin is the only known substrate for LIMK, and LIMK1 was demonstrated to be a direct modulator of actin polymerization (131). These results drew much attention from researchers. Vorster et al. corroborated that LIMK1 is involved in the early stages of HIV infection, as they observed that within 1–3 min after HIV gp120 binding to CXCR4, rapid LIMK activation in resting CD4^+^ T cells occurred (132).

In addition to the LIMK1-cofilin signaling pathway, the WAVE2–Arp2/3 signaling pathway also regulates actin activity during HIV infection (126). Rac belongs to the Rho family, and it is particularly important in the regulation of HIV fusion. Arp2/3 is a complex downstream of Rac that polymerizes actin (133, 134). The major role of the Arp2/3 complex is to regulate actin branching and nucleation, and which is directly activated by WAVE2 (135). The WAVE2–Arp2/3 signaling pathway is involved in the early steps of HIV infection (126). Harmon et al. proved that HIV Env-mediated fusion and entry were WAVE2–Arp2/3-signaling dependent (136). In brief, HIV gp120 binds to CD4 and a co-receptor to active GTPases in the Rho family, which further affects the actin cytoskeleton and promotes many processes of HIV infection (137). The cortical actin activation modulated by the two signaling pathways is important event in the initiation of HIV infection.

In line with the activation of actin induced by the two signaling pathways mentioned earlier, several inhibitors of the proteins involved in the pathways have been demonstrated to reduce HIV infection, such as Slit2N (124), R10015 (125), CK548 (126), and an Abl kinase inhibitor (127). Slit2, a ~200 kDa secreted glycoprotein, can regulate immune functions and inhibit the migration of various immunocytes induced by chemotactic signals (138, 139). Its N-terminal fragment (approximately 120 kDa), termed Slit2N, can bind to Robo1 to inhibit gp120/co-receptor-induced cytoskeletal rearrangements associated with LIMK- and cofilin-regulated actin polymerization in activated and resting CD4^+^ T cells (124). Moreover, R10015, a small-molecule LIMK inhibitor, can inhibit viral DNA synthesis, nuclear migration, and virion release (125); CK548 can inhibit the Arp2/3 complex and decrease the branching of actin filaments (126, 140); and Abl kinase inhibitor can inactive WAVE2 to inhibit HIV entry (127). These inhibitors should be further studied with regard to their potential clinical applications.

### Effect of Other Molecules on Actin-Related Signaling Pathways

Proteins in the ezrin–radixin–moesin (ERM) family have been widely studied as regulators of cancer progression-related signaling that are involved in cell adhesion, migration, and polarity (141). ERM proteins appear to act as cross-linkers between the plasma membrane and actin (142). Moesin, a member of the ERM family, is not only correlated with cancer progression, but also plays a key role in HIV infection (143). During the early steps of HIV infection, phosphorylated (activated) moesin induces actin filaments to attach to the plasma membrane, which is necessary for CD4 and co-receptor CXCR4 clustering and interaction (144). Moesin facilitates HIV adhesion and entry during the very early steps of HIV infection. Once gp120 binds to CD4, moesin-induced actin redistribution and rearrangement at the plasma membrane leads to CD4 and CXCR4 interaction and co-localization, which lay the foundation for subsequent fusion and entry of the HIV virus (118, 119). In addition, moesin also acts as a nucleation factor for F-actin polymerization (144). Given that moesin plays important roles in HIV infection, decreasing its activity may be useful for inhibiting HIV invasion (144).

Filamin-A, an adaptor protein that can link the actin cytoskeleton to HIV receptors, is another factor that affects HIV invasion due to its functional role in RhoA-dependent signaling pathway activation. Filamin-A phosphorylates cofilin (thereby activating it), and further organizes the arrangement of F-actin, which may facilitate HIV infection (120). As a consequence, regulation the activity of filamin-A could affect HIV invasion. Gelsolin is an actin adaptor with filament-severing activity, and overexpressing and silencing gelsolin have been shown to impair efficient HIV fusion and infection due to disruption of cortical actin (121). At the same time, overexpressing and silencing gelsolin impairs gp120-induced actin rearrangement and viral receptor capping. As a result, regulating either the gelsolin expression level or its filament-severing activity might be useful strategies for fighting HIV (121).

## High Levels of Chemokines Impair Lymphocyte Functions

In both acute and chronic HIV infection, the alteration of chemokine levels benefits HIV infection and latency. In addition to these effects on HIV, elevated chemokines also impair the immune system by directly suppressing immune cell functions. For instance, high levels of plasma CXCL10 suppress T cell function in HIV-infected subjects (56). Our previous study suggested that CXCL10 could also suppress NK cell function by binding to CXCR3 during HIV infection (145). Innate and adaptive immune cell dysfunction may severely impact HIV transmission and disease progression. Lane et al. demonstrated that CXCL10 treatment led to increased HIV DNA accumulation in monocyte-derived macrophages and peripheral blood lymphocytes (146). Adaptive and innate immunity are both influenced by CXCL10, to different degrees, during HIV infection. More attention should be paid to the high levels of multiple chemokines, which may cause immune system dysfunction.

The frequency of CXCR3^+^ and CCR6^+^ central memory T cells in blood was demonstrated to be positively correlated with soluble plasma CD14, a marker of chronic systemic immune activation (147). In other words, chronic immune activation, induced by chronic HIV infection, might contribute to the accumulation of these cells in the peripheral blood. Moreover, persistent immune activation could cause the impairment of the CCR6^+^ and CXCR3^+^ Th cell response to CCL20 and CXCL10, preventing the migration of the Th cells to lymphoid organs (147). As more HIV DNA has been detected in CCR6^+^ and CXCR3^+^ cells, the disrupted migration of these susceptible cells might be responsible for the prevention of HIV clearance. However, by exploring the underlying mechanism, researchers have revealed that impairment of cell migration during HIV infection is at least in part due to the hyperactivation of cofilin and inefficient actin polymerization (147). Overall, inhibition of chemokines by antibodies or antagonist might protect immune cell function and help to increase the ability of the immune system to control virus by itself.

## Conclusion

When HIV invades the human body, it uses multiple mechanisms to ensure its survival and reproduction. However, the host immune system presents various barriers to make virus survival and reproduction difficult. The cytoskeleton is composed of cortical actin not only maintains the cell shape but also acts as a physiological barrier to pathogens. Generally, it is difficult for pathogens to traverse the cross-linked actin, but HIV utilizes gp120 to bind CXCR4 or CCR5, thereby mimicking signaling induced by their respective ligands. As a result of the activation of the signaling pathway, the cortical actin is rearranged, which makes the cell “open the door” to HIV. Regardless of whether the HIV infection involves the early and late phase, persistent inflammation occurs, with high levels of cytokines (including chemokines). This disrupts the immune system’s fight against the virus, thereby favoring the virus. Chemokines may activate chemotaxis signaling pathways, which further activate actin and allow HIV to infect cells more easily. Multiple chemokines elevated during HIV infection, independent of gp120, are enough to activate the actin-related signaling pathway to promote HIV infection and latency. The chemokines also suppress the function of innate and adaptive immune cells and prevent cell migration and homing, all of which decrease viral clearance. In essence, chemokines and chemokine receptors serve as accomplices to HIV during HIV infection and latency. However, only a few chemokines had been studied in HIV infection; other relevant chemokines remain unresearched and need to be further studied in terms of HIV pathogenesis and signaling pathway targets for prevention and treatment.

The existence of HIV reservoirs remains a major obstacle to curing HIV, and chemokines and chemokine receptors might influence its establishment, suggesting that they might be promising therapeutic targets. The related mechanism regarding the effect of actin on HIV infection and latency was discussed, along with several small-molecule drugs that have been shown to efficiently inhibit HIV fusion, entry, integration, and release, thereby providing new immune therapy strategies against HIV infection.

Thus, we put forward a therapeutic strategy based on HIV pathogenesis related to chemokines and chemokine receptors. At the start of an HIV infection or at ART withdrawal (as immune reconstruction occurs), patients should be treated with antibodies against certain chemokines, chemokine receptor antagonists or inhibitors of the actin-related signaling pathway, to suppress HIV replication and control rebound virus by enabling a functional immune response, and ultimately to achieve a functional cure.

## Author Contributions

ZW and YJ wrote the manuscript and drew the figure. HS revised the manuscript. All the authors revised the manuscript and approved it for publication.

## Conflict of Interest Statement

The authors declare that the research was conducted in the absence of any commercial or financial relationships that could be construed as a potential conflict of interest.

## Abbreviations

HIV, human immunodeficiency virus; AIDS, acquired immunodeficiency syndrome; ART, antiretroviral therapy; GPCR, G protein-coupled receptor; NK, natural killer; SIV, simian immunodeficiency virus; DC, dendritic cell; APOBEC3A and APOBEC3G, apolipoprotein B mRNA-editing enzyme-catalytic polypeptide-like 3A and 3G; hBD2, human β-defensin 2; MDM, monocyte-derived macrophage; PBMC, peripheral blood mononuclear cell; ERM, ezrin–radixin–moesin; F-actin, filamentous actin.
